# Prospective Evaluation of Dental Practitioners' Knowledge, Attitude, and Practice Toward Adult Dental Pain Management: A Cross-Sectional Multicenter Study

**DOI:** 10.7759/cureus.55388

**Published:** 2024-03-02

**Authors:** Kirti Shukla, Kranthi Kiran Pebbili, Seema V Bhagat, Rahul Rathod, Bhavesh P Kotak

**Affiliations:** 1 Medical Affairs, Dr. Reddy's Laboratories Ltd, Hyderabad, IND; 2 Medical Affairs, Dr. Reddy’s Laboratories Ltd, Hyderabad, IND

**Keywords:** india, analgesics, knowledge-attitude-practice theory, ketorolac, adult dental pain management

## Abstract

Background

Dental pain among adults is a prevalent concern impacting oral health and quality of life. Dental pain management presents a significant challenge for dental practitioners in effectively alleviating patient discomfort. Among the medications available, non-steroidal anti-inflammatory drugs (NSAIDs) are considered the most effective analgesics in dental care. While numerous studies have been conducted to assess the role of antibiotics in dental practice, there is a scarcity of studies specifically examining the prescription patterns of analgesics. The primary aim of this study was to evaluate the knowledge, attitudes, and practices of dental practitioners (DPs) in India concerning the management of adult dental pain.

Method

This survey utilized a computer-aided questionnaire-based approach. A total of 110 dentists, including 16 from metropolitan areas and 84 from non-metropolitan cities practicing at dental healthcare setups, clinics, and hospitals, were interviewed as part of the survey. The participants comprised dental professionals specializing in prosthodontics, endodontics, orthodontics, periodontics, oral surgery, pedodontics, and oral medicine. The study was conducted between September 2022 and January 2023.

Results

The primary reason patients seek dental consultation, as reported by 95% of dentists, is tooth cavities, followed by tooth sensitivity, post-root canal treatment, and pulpitis. All surveyed dentists prescribed NSAIDs to their patients for managing dental pain. Local anesthesia (LA) was the second choice for 75% of dentists, prescribed to 23% of their patients. The primary use of NSAIDs was for patients experiencing severe pain and to manage post-procedure pain. Eighty percent of DPs recognized ketorolac as a fast-acting molecule, providing immediate relief within 10-15 minutes. Overall, analysis indicated that 98% of DPs are satisfied and 67% are extremely satisfied with ketorolac among monotherapies for dental pain management due to its quick onset of action, fast pain relief, and usefulness in post-surgical pain management.

Conclusion

NSAIDs like ketorolac, diclofenac, and aceclofenac were the preferred prescriptions for overall dental pain management. Dental practitioners associated ketorolac with fast pain relief, quick onset of action, and effectiveness in post-surgical pain management, emphasizing its lasting effects. The insights from the study contribute to enhancing dental pain management strategies.

## Introduction

Dental pain, or odontalgia, is the most frequent complaint among dental patients suffering from dental diseases or undergoing interventional procedures [1]. Prolonged episodes of pain impact the quality of life (QoL) of an individual and may progress to chronic pain, which further influences both the physical and mental health of the patients [2,3]. Therefore, the management of dental pain becomes crucial, particularly when it comes to selecting the appropriate pain relief therapy. Several prerequisites must be considered when choosing an effective, safe analgesic treatment, and one of these is the intensity of pain experienced by the patient. Additionally, the medical condition of the patient, potential drug-drug interactions, and the cost of treatment are also crucial factors to consider during the selection process [1,4].

The most prescribed medications in dental care include anti-inflammatory drugs (AIDs), analgesics, antibiotics, and antiseptics. Steroids and non-steroidal anti-inflammatory drugs (NSAIDs) are frequently prescribed for various dental conditions, such as dental or orofacial pain, postoperative pain, chronic pain, and dental implant surgery [5]. Among these medications, non-steroidal anti-inflammatory drugs are considered the most effective analgesics in dental care.

NSAIDs are often preferred over other analgesics as they provide effective pain relief, reduce inflammation, and have a shorter duration of treatment, making them suitable for a wide range of conditions. NSAIDs have a lower risk of dependence and addiction compared to opioid analgesics, making them a safer option for managing pain. The mechanism of action of NSAIDs involves inhibiting the activity of an enzyme called cyclooxygenase (COX). COX plays a role in the production of prostaglandins, which are chemical mediators that contribute to pain, inflammation, and fever. By inhibiting COX, NSAIDs reduce the production of prostaglandins, thereby alleviating pain and reducing inflammation [6-8].

Ketorolac is a preferred NSAID due to its efficacy, safety profile, and cost-effectiveness. It offers comparable analgesic efficacy to other NSAIDs, making it a suitable alternative for pain management [9-11].

While numerous studies have been conducted to assess the role of antibiotics in dental practice, there is a scarcity of studies specifically examining the prescription patterns of analgesics. The aim of this study was to assess current dental practice details and knowledge of dental pain, understand dental practitioners' perception towards pain management, and explore their attitude toward managing dental pain, including factors considered while assessing severity and choosing analgesics.

## Materials and methods

Study design, setting, and population

This was a questionnaire-based survey to determine the knowledge, attitude, and practices in dental pain management among dental practitioners in India carried out from September 2022 to January 2023.

Dental professionals with a Bachelor of Dental Surgery (BDS)/Master of Dental Surgery (MDS) degree specializing in prosthodontics, endodontics, orthodontics, periodontics, oral surgery, pedodontics, and oral medicine with a patient load of average 280 per month and having 12-13 years of experience were included in the study.

Sample size calculation and participant selection

A total of 110 dental practitioners were randomly selected from the list of 600 practitioners for the study using a simple random sampling technique. They were contacted through phone or mail and invited to participate. For the practitioners who agreed to participate, a 30-minute appointment was scheduled to explain the study details, obtain informed consent, and administer a questionnaire. The quantitative phase of the study involved 100 practitioners who completed one-on-one online interviews. Additionally, the qualitative phase included 10 practitioners who underwent in-depth telephonic interviews. Throughout the interviews, all discussions were recorded with the consent of the practitioners and later transcribed for analysis.

For the quantitative study, six Metro (Bangalore, Chennai, Delhi, Hyderabad, Kolkata, Mumbai) and a mix of non-Metro (mix of Tier 1, Tier 2, Tier 3 cities across India) cities were covered and for the qualitative study, two Metro (Bangalore, Mumbai) and a mix of non-Metro (Tier 1 - Lucknow, Pune, Tier2 - Vijayawada, Guwahati, Coimbatore, Tier 3 - Siddipet, Dindigul, Kolhapur) were considered.

Ethical considerations

All aspects of the study with protocol No. DRL-IND-GGI14-KAPD/2022 were managed in compliance with Good Clinical Practice (GCP) and all applicable regulatory requirements. This study was conducted in compliance with ethics committee requirements as per applicable regulations and also in compliance with the Declaration of Helsinki. All study documents were reviewed and approved by the Royal Independent Ethics Committee (IEC).

Statistical analysis

Descriptive analysis was used to understand the current state of knowledge, attitudes, and practices; correlation analysis was applied to reveal how these elements are related to each other; and regression analysis was used to predict how changes in knowledge or attitudes might affect practices.

## Results

Current practices

**Figure 1:**
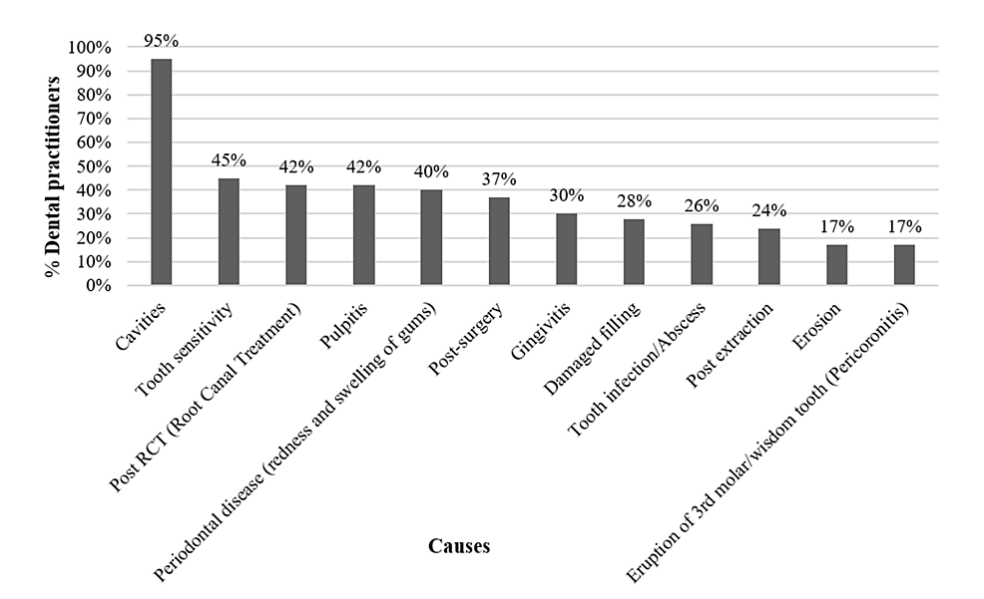
Causes of dental pain

Among the 110 dental practitioners, 71% (n=79) of dentists acknowledged that patients experience a diminished quality of life due to dental pain. Additionally, according to 85% (n=94) of dentists, patients seek their assistance for prompt and lasting dental pain relief, irrespective of the pain's severity. The primary reason patients seek dental consultation, as reported by 95% (n=105) of dentists, is tooth cavities, followed by tooth sensitivity, post-root canal treatment (RCT), and pulpitis (Figure 1).

Moreover, considering the severity of dental pain, all post-surgery patients (100%) choose to schedule a dental visit. This is followed by 83% of patients with severe pain after dental extraction and 81% of patients experiencing pericoronitis. The details are shown in Table 1.

**Table 1:** Dental visits according to severity of dental pain

Causes of dental pain	Severity of dental pain (%)		
	Mild	Moderate	Severe
Cavities	8	75	17
Tooth sensitivity	0	67	33
Post-RTC (root canal treatment)	3	35	62
Pulpitis	0	50	50
Periodontal disease (redness and swelling of gums)	0	71	29
Post-surgery	100		
Gingivitis	0	41	59
Damaged filling	0	41	59
Tooth infection/abscess	0	50	50
Post-extraction	0	18	83
Erosion	0	25	75
Eruption of 3rd molar/Wisdom tooth (pericoronitis)	5	14	81
Root fracture	0	35	65
Abrasion	0	21	79
Tooth fracture	0	47	53
Attrition	0	67	33
Dentoalveolar fracture	9	36	55
Untreated infection	0	50	50
Damage of temporomandibular joint (TMJ)	0	58	42
Tooth intrusion/avulsion	0	49	51

Diagnostic test for dental pain

It was found that 55% (n=61) of dentists confirmed performing both clinical assessments and diagnostic tests to determine the cause of dental pain, while 29% (n=32) recommend only the diagnostic tests. Among dentists who recommend diagnostic tests alongside clinical assessment, the most recommended diagnostic test was the intraoral periapical radiograph (n=94, 85%), followed by palpation (n=77, 70%) and orthopantomogram (n=72, 65%; Table 2).

**Table 2:** Diagnostic test recommended for dental pain

Diagnostic test	Dentists' preference (%)
Iintraoral periapical (IOPA) radiograph	85
Palpation	67
Panoramic orthopantomography (OPG)	65
Mobility test	46
Percussion	45
Complete blood count (CBC)	39
Water test - hot and cold	31
Blood tests	25
Air blast	12

Treatment goals

According to the data captured from one-on-one, in-depth telephone interviews, patients prioritized immediate pain relief as the most crucial aspect, while dentists also considered it a significant treatment objective. Although treatment goals generally remain consistent across different age groups and levels of severity, the treatment plans tend to vary for elderly patients depending on the severity of the condition. All dentists (n=110, 100%) prescribed NSAIDs to their patients for managing dental pain. Local anesthesia (LA) was the second choice for 75% (n=83) of dentists, prescribed to 23% (n=65) of their patients. The primary use of NSAIDs was for patients experiencing severe pain 85% (n=238) and to manage post-procedure pain 80%(n=224). A significant proportion of dentists (n=77, 70%) believed that NSAIDs play a crucial role in effectively managing post-procedure pain due to their anti-inflammatory properties.

Over 80% (n=88) of dental practitioners strive to achieve immediate pain relief, address swelling, and consider both the severity (mild, moderate, severe) and type of pain (throbbing, intense, persistent, intermittent) before determining the appropriate treatment. Other crucial factors considered before initiating treatment were the age of the patient, the duration of pain, and the safety of the prescribed molecules (Table 3). These factors were deemed the most important, followed by considerations of efficacy, fast pain relief, and any comorbidities present.

**Table 3:** Factors considered before prescribing any treatment

Factors considered	Dentists, %
Severity of pain (mild, moderate, and severe)	96
Type of pain (throbbing, intense, persistent, intermittent)	86
Age	79
Duration of pain	68
Safety of molecules for long-term use	67
Efficacy	63
Fast pain relief	62
Comorbidities (hypertension, diabetes, heart disease)	59
Frequency of pain	59
Duration of action	49
Onset of action	49

Preference of NSAID molecule in dental pain management

According to the survey results, NSAIDs are the preferred therapies recommended by dentists for managing various types of dental pain. The use of NSAIDs is beneficial as they possess anti-inflammatory properties that help reduce both pain and inflammation. Among the most prescribed NSAIDs, combinations such as aceclofenac + paracetamol + serratiopeptidase and aceclofenac + paracetamol are often used in cases where pain is accompanied by swelling.

The survey revealed that ketorolac was the most frequently prescribed NSAID by 70% (n=77) of dentists in cases of moderate and severe pain during monotherapy, and these dentists reported high satisfaction with its usage. Among the NSAIDs, ketorolac is predominantly prescribed by DPs (n=77, 70%), followed by diclofenac (n=72, 65%) and aceclofenac (n=66, 60%) for monotherapy in dental pain management. Ketorolac is administered across all phases of dental pain and is the most preferred option among the analgesics (monotherapy/combinations) by DPs. In contrast, diclofenac and aceclofenac were primarily used among patients with mild pain (n=34, 31%, and n=33, 30%, respectively; Table 4).

**Table 4:** NSAIDs prescription for dental pain management NSAIDs - non-steroidal anti-inflammatory drugs

NSAIDs	Severity of pain, n		
	Mild	Moderate	Severe
Ketorolac	29	37	43
Diclofenac	31	20	30
Aceclofenac	30	25	22
Paracetamol + aceclofenac	30	33	32
Acetaminophen/ paracetamol	29	23	21
Ibuprofen	28	27	21
Piroxicam	24	27	24

Ketorolac prescription in dental pain management

From the qualitative phase of the survey, eight out of 10 (80%) DPs recognized ketorolac as a fast-acting molecule that provided immediate relief within 10-15 minutes. It is primarily preferred for young to middle-aged patients (between 20-55 years) experiencing acute dental pain, particularly in cases of moderate to severe pain.

In the quantitative survey of 100 dentists, 40% of DPs mentioned that ketorolac has a relatively short duration of action, with its impact lasting for four to six hours. In terms of prescription patterns, ketorolac was prescribed by 38% (n=38) of DPs in the post-dental procedure stage, closely followed by 31% (n=31) of DPs during dental procedures. Typically, ketorolac was prescribed twice a day for three to five days in various indications of moderate to severe dental pain, such as post-tooth extraction, RCT, and other dental surgeries. In RCT specifically, ketorolac was also prescribed as needed (SOS) by 27% (n=27) of DPs.

**Figure 2:**
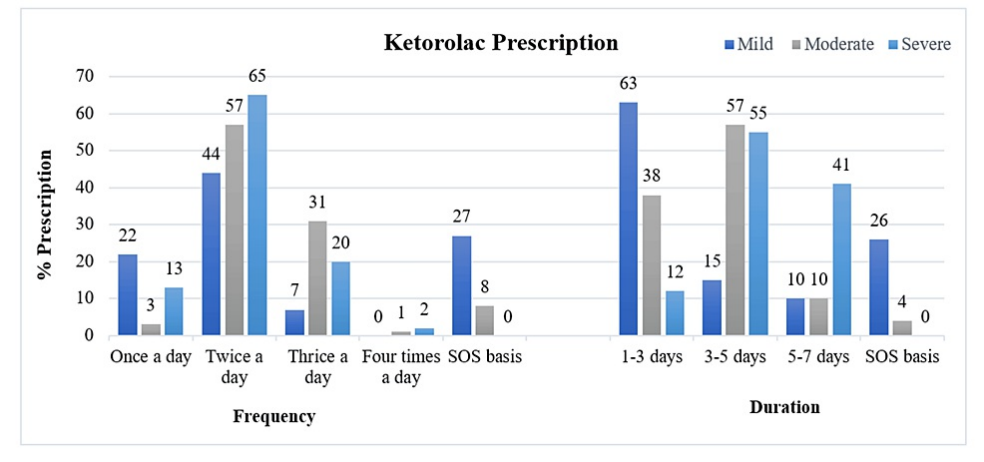
Ketorolac prescription

The prescription duration displayed variation based on pain intensity. For cases of mild pain, 44% (n=40) of dentists prescribed a ketorolac BD dose, with 63.6% (n=70) of dentists recommending a duration of one to three days. On the other hand, for patients with severe pain, 65% (n=72) of dental practitioners prescribed ketorolac BD, while 41% suggested a longer duration of five to seven days. Approximately 55% (n=61) of dentists opted for a duration of three to five days for severe pain cases. Most dentists, 96%(n=106), prescribe ketorolac for three or more days for severe dental pain, while 60% (n=66) of dentists prescribe ketorolac for three or more days for moderate pain. On the other hand, 87% (n=96) of dentists prescribe ketorolac two or more times a day for severe dental pain, while 89% (n=98) of dentists prescribe ketorolac two or more times a day for moderate pain. The most preferred dose for moderate to severe pain for ketorolac was twice or more daily for three to five days (Figure 2).

Overall, the analysis indicated that 98% (n=108) of DPs are satisfied and 67% (n=74) are extremely satisfied with ketorolac among monotherapies for dental pain management due to its quick onset of action, fast pain relief, and usefulness in post-surgical pain management.

## Discussion

NSAIDs are commonly prescribed by dentists for pain relief following tooth extractions and as postoperative analgesics [12]. Unlike opioids, NSAIDs offer the benefits of alleviating mild-to-moderate dental pain and reducing inflammation. A detailed survey was carried out to understand the perspectives and practices of dental practitioners in India regarding pain management. This survey sought to explore their knowledge, attitudes, and practice patterns towards NSAIDs and pain relief strategies.

The survey revealed that 85% of patients experiencing severe pain were prescribed NSAIDs. Dentists reported that dental pain adversely affects the QoL of patients who seek medication regardless of the pain's intensity. There is a clear preference among patients for treatments that offer rapid and enduring relief from pain.

Before commencing any therapeutic intervention, a precise diagnosis of the condition is essential. According to the data collected in our survey, 55% of dentists utilized a combination of clinical evaluations and diagnostic tests to prescribe medications, whereas 29% relied solely on diagnostic tests for the verification of dental pain. These results align with the findings of a study by Monisha et al., conducted in India, which reported that 63% of practitioners based their pharmacological prescriptions on diagnostic outcomes [4]. Preferred diagnostic modalities among respondents included intraoral periapical (IOPA) radiography, palpation, and orthopantomogram (OPG) scans. The survey underscores the necessity of aligning the pharmacological characteristics of medications with the diagnosed conditions to achieve targeted and efficacious treatment outcomes.

The research conducted by Dionne and McCullagh underscored the efficacy of NSAIDs in reducing inflammation-induced swelling [13]. Their findings specifically pointed to ibuprofen's capability to modulate neurohumoral responses, thereby effectively diminishing swelling. This is supported by various studies that advocate for NSAID use as a proactive strategy to lessen the intensity of pain and postpone its commencement [14,15]. Data from our survey indicated a significant preference among dental practitioners for specific NSAIDs: approximately 70% favored ketorolac, 65% chose diclofenac, and 61% selected aceclofenac for dental pain management. Contrastingly, a separate study in India identified diclofenac as the preferred NSAID for 50% of the surveyed practitioners [4].

The safety and efficacy of ketorolac have been supported by several studies, as it does not impede the central respiratory drive or cause gastrointestinal discomfort [16,17]. Moreover, in our study, ketorolac was preferred because safety is less of a concern when compared to other NSAIDs when managing severe dental pain. Although ibuprofen is effective in reducing postoperative dental pain, its prescription is limited due to poor tolerability. In our study, ibuprofen was preferred by only 23% of patients for dental pain relief.

Hence, it is evident that the pharmacological properties of a drug play a critical role in treatment selection. The choice of medication is influenced by factors such as its effectiveness, safety profile, and tolerability, which ultimately guide dental practitioners in their decision-making process [4].

Furthermore, our observations revealed that the predominant prescription of ketorolac occurred in postoperative scenarios. Research studies have substantiated that the preoperative administration of ketorolac leads to a substantial reduction in operative pain, initial postoperative pain and even delays the onset of postoperative pain [18]. Another study by Fricke et al. emphasized that ketorolac serves as a viable alternative to opioids and other NSAIDs in managing moderate-to-severe postoperative surgical pain, offering superior relief and prevention [19].

When prescribing ketorolac, it is essential for practitioners to consider any pre-existing medical conditions of the patient. It is generally recommended to limit the prescription duration to a maximum of five days [19]. In line with this, our survey also revealed that ketorolac was typically prescribed twice daily for a duration of three to five days in cases of moderate pain. 

Strength of the study

The study's strength lies in its comprehensive evaluation of the current understanding, perspectives, and practices of dental practitioners in India regarding pain relief management, with a particular focus on NSAIDs. By conducting a wide-ranging survey, the research offers valuable insights into the prevalent prescribing habits for managing dental pain, highlighting the preference for NSAIDs like ketorolac.

Limitations of the study

Despite the numerous advantages of the study, it is important to recognize that there are certain limitations. Firstly, the cross-sectional design restricts the ability to establish causality and only provides a snapshot of the participants' knowledge, attitudes, and practices at a single point in time. Secondly, the study's findings may be subject to selection bias, as the sample may not represent the entire population of dental practitioners in India. Additionally, the reliance on self-reported data may introduce response bias, due to the socially desirable answers provided by the participants or overestimate their knowledge and practices. Additionally, while the study provides insight into the preferences and practices of dental practitioners in India, its findings may not be universally applicable to different geographic or cultural contexts, limiting the generalizability of the results. Furthermore, the study does not extensively explore the potential side effects or limitations of NSAID use in certain patient populations, which is crucial for a holistic understanding of pain management in dental practice.

## Conclusions

This study on knowledge, attitudes, and practice reveals important insights into current practices and preferences of dental practitioners in managing dental pain. NSAIDs, particularly ketorolac, diclofenac, and aceclofenac, were the preferred prescriptions. Ketorolac was prescribed for all pain severity levels, primarily in moderate to severe cases, with a frequency of twice daily for three to five days. Dental practitioners associated ketorolac with fast pain relief, quick onset of action, and effectiveness in post-surgical pain management, emphasizing its lasting effects. The findings from this survey can guide future research and contribute to the development of more targeted and effective treatment strategies for dental pain management.
